# Impact of Milk Thistle Cake as the Natural Antioxidant Source on the Mitigation of Oxidative Effects in Goat Milk Induced by Oxidized Linseed Oil

**DOI:** 10.3390/foods14183205

**Published:** 2025-09-15

**Authors:** Alexandra-Gabriela Oancea, Catalin Dragomir, Petru Alexandru Vlaicu, Iulia Varzaru, Mihaela Saracila, Ana Elena Cismileanu, Mihail Alexandru Gras, Mircea Catalin Rotar, Arabela Elena Untea

**Affiliations:** National Research and Development Institute for Animal Biology and Nutrition, Calea Bucuresti, No. 1, 077015 Balotesti, Romania; alexandra.oancea@ibna.ro (A.-G.O.); catalin.dragomir@ibna.ro (C.D.); alexandru.vlaicu@outlook.com (P.A.V.); iulia.maros@ibna.ro (I.V.); mihaela.saracila@ibna.ro (M.S.); ana_cismileanu@yahoo.com (A.E.C.); mihai.gras@ibna.ro (M.A.G.); catalin.rotar@ibna.ro (M.C.R.)

**Keywords:** fat degradation parameters, linseed oil, milk thistle, milk antioxidant potential, milk fatty acids

## Abstract

This study explores a novel feeding strategy in the nutrition of dairy goats, utilizing milk thistle cake supplements to mitigate milk oxidation determined by the presence of oxidized linseed oil in diets. An experimental trial involving 30 dairy goats was conducted with three groups: a control group fed a diet with 7% fresh linseed oil (CON), an experimental group fed a diet where the fresh linseed oil from the CON group was replaced with oxidized linseed oil (LOO), and an experimental group fed a diet with 7% oxidized linseed oil and 10% milk thistle cake (LOM). The milk thistle cake had a rich antioxidant composition (vitamin E, xanthophylls, and polyphenols) with potential beneficial effects on milk degradation parameters. The results showed that the LOM diet led to a decrease in milk casein content (*p* = 0.041) while positively influencing the concentration of iron (13.24 vs. 14.93 mg/kg). In terms of fatty acids, the results suggested that milk thistle cake can counteract the negative effects of the oxidized oil (increasing SFAs, decreasing PUFAs and MUFAs) by modulating the content and reducing its negative effects. Moreover, the LOM group positively influenced the milk antioxidant potential by increasing the levels of antioxidant compounds (vitamin E, *p* < 0.001; total polyphenols, *p* < 0.01; antioxidant capacity, *p* < 0.0001). Moreover, an improvement in the milk primary and secondary degradation parameters was observed, i.e., a significant decrease in the levels of conjugated dienes (*p* = 0.023) and p-anisidine (*p* < 0.0001). The study demonstrated the benefits of using milk thistle cake in goat nutrition as it helps reduce the oxidative effects induced by oxidized linseed oil on the nutritional quality of milk and its degradation.

## 1. Introduction

Over the years, ruminant management has faced significant challenges in improving milk composition and nutritional quality, especially the fatty acid content [1,2]. Due to the rapid growth in animal production and the increasing human population, animal husbandry is facing ongoing global challenges concerning the shortage of animal feed resources. This situation has resulted in competition between feed and human food resources, leading to increases in feed prices and an urgent need to explore alternative feed sources [3]. Animal feeding strategies involving alternative feeds are supported by international organizations such as the Food and Agriculture Organization (FAO), which has strongly underscored the importance of designing nutrition for health by recycling and reusing by-products from agro-industries [4]. These strategies could counteract the rising cost of traditional feedstuffs while also supporting environmental sustainability. The process of valorizing by-products from various industries not only helps to reduce waste and pollution, but could also reduce feed costs, minimize competition with human food, and support ecological balance while improving the quality of animal-derived food products in a sustainable manner, as these by-products are rich in bioactive compounds [5]. Moreover, losses and waste from the food industry can be integrated into livestock nutrition, as they are considered “up-cyclers”. This approach plays an important role in reducing food waste by valorizing inedible food (i.e., used oil waste from the food industry, with a moderate level of oxidation that does not affect animal performance) and converting it into high-quality food products (meat, eggs, or dairy products) [5]. These alternative feeding strategies contribute to a reduction in feed costs and environmental burden while enhancing the nutritional composition of ruminant diets and the quality of dairy products, thereby supporting the principles of the 3Rs (reduce, recycle, reuse) [6].

In goat nutrition, one of the most effective strategies for enhancing the fatty acid profile of milk is through the incorporation of fat sources (oils and/or oilseeds) into their diets. Several studies have identified linseed oil as a promising feed component that can enhance the quality of the milk’s fatty acid profile [7,8]. Linseed oil is considered a valuable nutritional source of PUFAs, with the fatty acid composition comprising more than 47.5% linolenic acid, 20.5% linoleic acid, and only 9.65% saturated fatty acids. This exceptional polyunsaturated composition makes linseed oil a key factor that can enhance the fatty acid profile of goat milk [9]. This approach may reduce the levels of saturated fatty acids (SFAs), which are linked to various human diseases, while increasing the proportion of polyunsaturated fatty acids (PUFAs), which can benefit human health [10]. Recently, a study reported that although including 2.5% linseed oil in goats’ diet led to an improvement in C22:6n-3 concentration, a mixture of 4.16% linseed and fish oil was more efficient in influencing the content of health-promoting fatty acids, with no effects on milk production [11]. According to Kholif et al. [12], linseed oil is more effective than linseed meal in goats’ diet to increase milk production and improve the milk’s fatty acid profile, especially by increasing the proportions of conjugated linolenic acid (CLA) and overall total unsaturated fatty acids (UFAs) and decreasing the saturated fatty acid (SFA) concentration. In contrast, Liu et al. [13] reported that flaxseeds (4.80%) were more effective than flaxseed oil (2%) in improving the lipid profile of blood, and could potentially decrease the lipid deposition in goats. However, according to Almeida et al. [14], the type of oil (linseed, soybean, or fish oil) can influence different fatty acids, which, in turn, affect the goats’ milk and blood parameters. Linseed oil has effects comparable with those of fish oil, i.e., by improving the lipid profile of blood plasma, and the inclusion of linseed oil in milk enhances the content of eicosapentaenoic acid (EPA) and docosahexaenoic acid (DHA), which are very important for consumers’ health.

However, a less debated issue concerning these fat sources in ruminants, which have a significant impact on milk quality and animal health, is the susceptibility of dietary oils to the oxidation process. Under normal conditions, ruminants can naturally balance the production of free radicals resulting from cellular metabolism with their endogenous antioxidant capacity, which prevents the accumulation of these radicals. However, specific dietary conditions, such as using oxidized oil in their feed, can lead to an overload of free radicals, resulting in oxidative stress, which can negatively affect the performance of animals and economically impact the farmers [15]. Moreover, oxidized fats in ruminants can have direct effects on human health by altering the quality of fatty acids in milk and producing harmful oxidative by-products that are consumed by humans [16]. Therefore, the addition of antioxidants to animal diets has emerged as a strategy for increasing the commercial value of milk. Although synthetic antioxidants, such as butylated hydroxytoluene (BHT) and butylated hydroxyanisole (BHA), are widely used in animal nutrition to prevent lipid oxidation, consumer concern over their safety and toxicity has led researchers to find alternative natural sources of antioxidants [17,18].

Milk thistle (*Silybum marianum*) is a part of the *Asteraceae* family and an annual or biennial type of plant with a significant protein content, which has a major influence on ruminant nutrition [3]. It is a rich source of natural antioxidants, especially polyphenols and tocopherols, with important applications in animal nutrition regarding oxidative status [19]. The active substances in milk thistle are mainly found in its seeds [20]. Usually, the oil is extracted for therapeutic use in treating several human conditions [21]. However, after oil extraction, milk thistle cake is produced as a by-product rich in bioactive substances, which can be economically valorized in animal nutrition [20]. It contains a considerable amount of protein (about 23%), significant fat composition (26%), and an amount of carbohydrates and crude fiber of about 60%, which makes it an excellent feed source for ruminants. In addition, milk thistle cake contains a remarkable number of fatty acids, with a high content of linoleic and oleic acids ranging from about 49.8 to 60.32 % and 19.7 to 28.5% respectively, depending on the species [22]. The literature regarding the effects of milk thistle cake in dairy goats’ diets on milk quality is scarce. However, in peripartum dairy cows, beneficial effects were reported during the transition period [23,24]. In dairy goats, using 10, 20, and 30 g of milk thistle extract/day has been reported to improve the antioxidant status of the animals [25], enhance the digestibility of nutrients, and improve growth performance [26].

The use of milk thistle cake in ruminant nutrition requires caution due to concerns about oilseed cake quality. Contamination by fungi and mycotoxins, along with the oxidation of residual oils, can compromise safety and nutritional value. Therefore, strict quality control measures, including monitoring for fungal contamination and ensuring proper processing and storage, are essential to maintain the nutritional benefits and safety of milk-thistle-derived feed materials [27,28].

To the best of our knowledge, no studies in the literature have proposed a complex experimental design, specifically the use of oxidized linseed oil as a sustainable nutritional approach in dairy goat nutrition, nor the use of a natural source of antioxidant compounds to counteract the harmful effects of the oxidation process in milk.

Therefore, the objective of this study was to investigate the effects of milk thistle cake supplements to mitigate milk oxidation determined by the presence of oxidized linseed oil in the goats’ diet, and its effects on the nutritional quality of milk, as an innovative and sustainable nutritional strategy in dairy goat feeding.

## 2. Materials and Methods

### 2.1. Ethical Statement, Experimental Design, Diets, and Sample Collection

#### 2.1.1. Ethical Statement

The feeding trial was conducted according to relevant legislation (Directive 2010/63/EU) [29], and all procedures received approval from the Ethical Commission of the National Research and Development Institute for Biology and Animal Nutrition, Balotesti, Romania. The studied milk thistle cake was sourced from a local producer in Southern Romania.

#### 2.1.2. Experimental Design and Diets

The dietary experiment involved 30 multiparous goats of the Murciano-Granadina breed, with an average live weight of 44.50 ± 4.58 kg and an average age of 5.2 ± 1.19 years. At the start of the trial, the goats averaged 112.57 ± 12.11 days in milk, and the groups (114.00 ± 11.28—CON; 111.35 ± 12.44—LOO; and 112.37 ± 12.61—LOM) did not differ significantly in this parameter. The goats were also balanced with respect to the type of birth, with 15 single-born and 15 twin-born goats distributed evenly among the three groups. The goats were allocated into three groups; each being housed in collective paddocks depending on the specifics of each experimental batch. The experimental groups consisted of a control group (CON), fed a compound feed based on fresh linseed oil as a fat source (7%); the LOO group, fed a diet where the fresh linseed oil from the CON group was replaced with oxidized linseed oil; and the LOM group, fed a diet with oxidized linseed oil (7%) and milk thistle cakes (10%) as a natural antioxidant source. The goats had constant access to fresh water. The period of the nutritional experiment lasted 30 days, and before the dietary trial, all of the goats involved in the experiment had a two-week adaptation period. The milk was sampled in the last week of the experimental period. All three diets were formulated to be balanced regarding energy and nitrogen supply, using intestinally digestible protein (IDP) and milk feed units (MFUs) as the main parameters [30]. The administration of the compound feed was limited to 1 kg/head/day. The structure and proximate composition of the experimental diets are presented in Table 1.

#### 2.1.3. Sample Collection

In the final week of the nutritional experiment, the milk was sampled from all goats (N = 30 milk samples; n = 10 milk samples/group) involved in the trial. The milk was collected using an automatic milking machine, and the samples were stored at −4 °C until the completion of the analysis.

### 2.2. Chemical Analyses

#### 2.2.1. Oxidation State of Linseed Oil

Fresh linseed oil was purchased from a local producer from Dâmbovița County. The oil was placed in an open container and heated in a stove at 100 C for 8 h. Moderate oxidized linseed oil was obtained and analytical methods were applied for evaluating the oxidation degree. To determine the oxidation state of the oils used in this experiment, the analysis of the peroxidation value was assessed, following the protocol reported by [30]. After dissolving the samples in a mixture of chloroform/methanol (7:3), xylenol orange (10 mmol/L) and FeCl_2_ (1000 mg/kg) were added. The resulting solution was kept in the dark for 5 min, following the recording of the absorbances at 560 nm. The results were expressed as oxygen milliequivalents/kg lipids (meq O_2_/kg).

#### 2.2.2. Proximate and Mineral Composition of Milk Thistle Cake and Milk Samples

The determination of the crude protein from vegetal material was performed using the Kjeldahl method, with a semiautomatic Kjeltek auto 1030-Tecator system (FOSS Tecator AB, Höganäs, Sweden). The crude fat concentration was assessed using Soxhlet determination with Soxtec 2055 Foss Tecator equipment (Höganäs, Sweden). The content of dry matter and ash was assessed using the gravimetric method with a BMT ECOCELL Blueline Comfort (Neuremberg, Germany) and a Nabertherm Labotherm L15/11/P320 Comfort (Bremen, Germany). The methods are described in [31].

For the determination of the milk proximate chemical composition (crude protein, crude fat, total caseins, lactose, density, and pH), a CombiScope FTIR 200 system (PerkinElmer, Waltham, MA, USA) was used, following the standard methods in this field (ISO 9622:2013) [32].

The mineral composition of the vegetal material and milk samples was obtained as follows. The content of copper, iron, manganese, zinc, and magnesium was studied by flame atomic absorption spectrometry (FAAS) using a Thermo Electron SOLAAR M6 Dual Zeeman Comfort system (Cambridge, UK) [33]. The calcium content was assessed by a complexometric method, using ethylenediaminetetraacetic acid (EDTA) as a titration agent in the presence of murexide. The analysis of phosphorus content was performed using a spectrophotometric method, employing molybdovanadate and a spectrophotometer Jasco V530 UV/VIS (Japan Servo Co., Ltd., Tokyo, Japan) [33].

#### 2.2.3. Hydro-Soluble Compounds of Milk Thistle Cake and Milk Samples

The total polyphenol (TP) content was studied via the Folin–Ciocalteu method. The technique was previously described in [31]. The extraction of the samples was performed using 1 g of dried samples mixed with 10 mL of methanol, with the sample kept in the dark for 24 h in a rotary shaker. Then, 0.5 mL of the extract was mixed with 0.5 mL Folin–Ciocalteu reagent, 7 mL water, and 20% (2 mL) sodium carbonate solution. After keeping the solution in the dark for one hour, the absorbance was measured at 732 nm. The gallic acid calibration curve was used for total polyphenol quantification, and the results were expressed as mg gallic acid equivalents/g of dried sample.

The polyphenol profile of the milk thistle cake was assessed by high-performance liquid chromatography using a Vanquish Core HPLC Instrument (Thermo Fisher Scientific, Bremen, Germany) liquid chromatograph with a BDS HyperSil C18 column (250 × 4 mm, 5 µm particle size, Thermo Fisher Scientific, Bremen, Germany). The mobile phase contained three solvents: 1% acetic acid (solvent A), methanol (solvent B), and acetonitrile (solvent C). The method was carried out using the following elution program: the mobile phase initially contained 5% solvent B and 5% solvent C. Between 15 and 20 min, the composition changed to 4% B and 15% C. From 20 to 25 min, it was adjusted to 3% B and 25% C. Between 25 and 40 min, the proportion was set at 2% B and 38% C. Finally, during the period from 40 to 50 min, the system returned to the initial conditions of 5% B and 5% C. The identification of phenolic compounds was assessed using reference standards of the individual polyphenols. The method was previously described in [31].

#### 2.2.4. Lipo-Soluble Compounds of Milk Thistle Cake and Milk Samples

The profile of fatty acids was obtained using a Perkin Elmer Clarus 500 gas chromatograph (Waltham, MA, USA) following the method described in [34]. A flame ionization detector (FID) and a highly polar capillary column, TRACE TR-Fame (Thermo Electron, Waltham, MA, USA), were used for determination with the following characteristics: 60 m × 0.25 mm × 0.25 µm film.

Fat extraction was conducted in order to determine the lipo-soluble vitamins (vitamin A and vitamin E isomers) and xanthophylls following the protocol described by Varzaru et al. [35]. For the analysis, 2 g of the sample was mixed in a conical flask with 130 mL ethanol, 100 mg butylated hydroxytoluene, 2 mL sodium ascorbate solution, 50 mg EDTA, and 25 mL potassium hydroxide (50%). After boiling in a water bath with a condenser for 30 min at 80 °C, the resulting solution was transferred into a separation funnel by rinsing with 250 mL of water. Then, the conical flask was rinsed with 100 mL petroleum ether and 25 mL ethanol and transferred to the separation funnels, and the extraction was repeated with petroleum ether four times. After washing the solution in the separation funnels with 100 mL of water, the resulting extract was filtered through anhydrous sodium sulfate and evaporated under vacuum conditions. The remaining residue was dissolved in 10 mL ethanol.

The assessment of the vitamin E (alpha-, gamma-, and delta-) isomers and vitamin E was performed using high-performance liquid chromatography with a Vanquish Core HPLC system (Thermo Fisher Scientific, Bremen, Germany). The column used was a C18 column (Thermo Fisher Scientific, Waltham, MA, USA), while the mobile phase consisted of two solvents: 96% methanol and 4% water [35]. The flow of the mobile phase was 1 mL/min. The results were expressed as mg/kg dried sample.

The xanthophyll content (lutein and astaxanthin) was determined using high-performance liquid chromatography. The system used was a Finnigan Surveyor Plus chromatograph (Thermo-Electron Corporation, Waltham, MA, USA) with a mobile phase consisting of 75% acetone, 15% methanol, and 10% water, and a mobile phase flow of 1 mL/min. The results were presented as mg/kg dried sample, and the protocol was previously described in [35].

#### 2.2.5. Antioxidant Activity of Milk Thistle Cake and Milk Samples

The total antioxidant capacity (AC) of milk thistle cake and milk samples was measured by a spectrophotometric method with a Jasco V-530 spectrophotometer (Japan Servo Co., Ltd., Tokyo, Japan) using the 2,2-diphenyl-1-picrylhydrazyl-hydrate (DPPH) method. The methanolic extract (0.4 mL) was mixed with DPPH 0.2 mM (2 mL) and water (1.6 mL), and vortexed. After keeping the solutions in the dark for 30 min, the absorbances were recorded at 517 nm with a Jasco V-530 spectrophotometer (Japan Servo Co., Ltd., Japan). The results were presented as millimolar (mM) Trolox equivalents per kilogram of sample [31].

#### 2.2.6. Fat Degradation Parameters of Milk Samples

The extraction of milk fat was performed using the modified Folch method, employing chloroform and methanol [31]. The milk samples (10 mL) were mixed in separation funnels with 60 mL chloroform, 30 mL methanol, and 7.5 mL KCl (0.88%). After separation, the lower layer was collected and evaporated at room temperature. In order to determine the degradation of the milk fatty acids, the primary (conjugated dienes, conjugated trienes, peroxidation value) and secondary degradation parameters (TBARS parameters and p-anisidine value) were studied. The determination of conjugated dienes (CDs) and conjugated trienes (CTs) was conducted following the method previously described by Untea et al. [31], using the lipid extract obtained in the previous step by the spectrophotometric method. The lipidic extract was mixed with iso-octane, and following this step, the absorbances were measured at 233 nm for CDs and 268 nm for CTs. The p-anisidine value was determined following the reaction between p-anisidine and the aldehyde compounds under acidic conditions [31]. The absorption of the iso-octane–lipidic extract solution was measured at 350 nm. Following this step, the p-anisidine reagent was added, and after 10 min in the dark, a new measurement at the same wavelength was performed. The p-anisidine value is conventionally defined as 100 times the change in absorbance measured at 350 nm, and the results are expressed in arbitrary units (A.U.). The determination of TBARS for the milk samples was conducted following the method previously described in [36], measuring the absorbance specific to milk fatty acid degradation at 450 nm (saturated aldehydes), 495 nm (dienals), and 532 nm (malondialdehyde). The milk sample (5 mL) was mixed with 20% trichloroacetic acid (10 mL) and centrifuged for 5 min at 3000 rpm. The resulting solution was incubated with thiobarbituric acid 0.8% for 90 min, and the absorbances at 450 nm, 495 nm, and 532 nm were recorded.

The spectrophotometric methods were performed using the Jasco V-530 spectrophotometer (Japan Servo Co., Ltd., Japan).

### 2.3. Statistical Analysis

The results of the proximate chemical composition and nutritional composition of milk were statistically analyzed by analysis of variance (ANOVA) with diet as the main factor, using XLStat by Addinsoft, version 2022.3.1 (New York, NY, USA), followed by Tukey’s post hoc test. For the milk degradation parameters (conjugated dienes, conjugated trienes) and TBARS parameters (absorbances detected at 450, 495, and 532 nm), the data was statistically analyzed using a bifactorial analysis of variance (storage time and diet as factors) (XLStat by Addinsoft, New York, NY, USA). The statistically significant differences were considered for the mean values with *p* < 0.05. The graphs were created using GraphPad Prism software version 9.3.0 (San Diego, CA, USA).

## 3. Results

### 3.1. Oxidation State of the Oils

**Figure 1:**
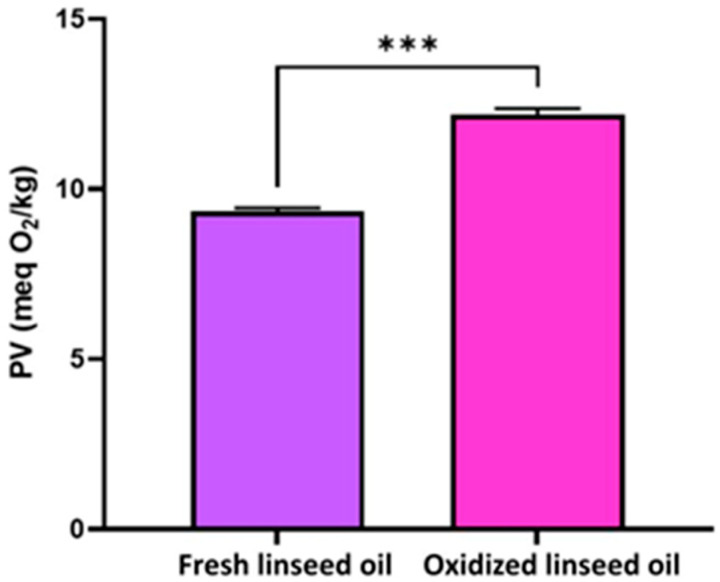
The peroxidation value (PV) of fresh and oxidized linseed oils. *** means significant difference at *p* < 0.001. The errors bars represented the standard deviations.

The oxidation state of the oils tested in the nutritional experiment was assessed through peroxidation value analysis. The results, presented in Figure 1, were used to evaluate the condition of the oils before they were added to the compound feed. The findings indicated that the oxidized linseed oil had a statistically significantly higher peroxidation value compared to the fresh linseed oil (*p* = 0.001).

### 3.2. Proximate, Mineral, and Lipid Composition of Milk Thistle Cake

Milk thistle cake represents an important source of protein, containing significant amounts of minerals and fatty acids (Table 2). Iron was the most abundant mineral, followed by zinc and manganese. The fatty acid profile showed that milk thistle cakes contain elevated levels of oleic and linoleic acids. Moreover, the samples exhibited a high mono- and polyunsaturated fatty acid content.

### 3.3. Bioactive Compounds of Milk Thistle Cake

Due to the high levels of unsaturated fatty acids determined in milk thistle cake, it was important to assess its antioxidant potential. Table 3 presents the results of its lipo-soluble antioxidant compounds (isomers of vitamin E and lutein), total polyphenol content, DPPH activity, and polyphenol profile. The results showed that milk thistle cake represents an important source of vitamin E, especially due to its high content of α-tocopherol among the isomers and notable amounts of lutein. In the case of individual polyphenols, milk thistle cake presented high content of phenolic acid, especially in the case of chlorogenic, syringic, protocatechuic, and ferulic acids. It also showed an impressive composition of flavonoids, particularly epicatechin, epigallocatechin, and rutin. The results also highlighted the notable antioxidant capacity of the studied milk thistle cake.

### 3.4. Influence of Dietary Treatments on Goat Milk Production and Composition

#### 3.4.1. Proximate and Mineral Composition of Goat Milk and Milk Production

The experimental diets had no significant effect on milk production monitored during the trial period (Table 4). A significant decrease in casein content was observed for the LOM milk samples (*p* = 0.041) compared with both CON and LOO. Similarly, the protein content presented a decreasing tendency (*p* = 0.054) for the LOM milk. The fat and lactose content were not influenced (*p* = 0.119, *p* = 0.273). However, the inclusion of the milk thistle cake in the goats’ diet led to a significant improvement in the iron content of the milk in the LOM group (*p* = 0.025) compared with the CON group. The phosphorus content was also significantly influenced (*p* < 0.031) by the experimental diets. No effect was observed for zinc and calcium among the groups.

#### 3.4.2. Lipid Profile of Goat Milk

The experimental diets significantly influenced the milk fat quality and the profile of fatty acids, as shown in Table 5. The milk fat quality was altered by the inclusion of the oxidized linseed oil in the goats’ diet. The LOO group exhibited a significantly higher content of SFA compared with the CON group. In contrast, the content of unsaturated fatty acids (MUFA, PUFA) was significantly reduced. On the other hand, the inclusion of the milk thistle cake in the group fed oxidized linseed oil counteracts its oxidative effects. As can be observed, the levels of saturated fatty acids tended to decrease, approaching the level detected in the control group, while the content of MUFA and PUFA increased, reaching values which not differ significantly from the CON. Also, it has to be highlighted that the LOM led to a significant increase in the conjugated linoleic acid (*p* = 0.008). The experimental diets did not significantly influence the cholesterol level of milk samples, but a numerical decrease in its content was observed for the LOM diet, compared with LOO and CON.

### 3.5. Antioxidant Potential of Goat Milk

Due to the influence of the experimental diets on the unsaturated fatty acid concentrations, it was necessary to quantify the lipo-soluble compounds with antioxidant properties in the milk matrix, as shown in Table 6. The lipo-soluble antioxidants in the milk were influenced by the experimental diets. The content of γ-tocopherol significantly increased in the LOM diet compared to both the CON and LOO diets. In the case of α-tocopherol, total vitamin E, and vitamin A, it was observed that the LOM diet showed intermediate values between the other two groups. The oxidized linseed oil reduced the concentrations of the studied lipo-soluble compounds, whereas the inclusion of milk thistle cake counteracted these effects, bringing their levels closer to those observed in the CON group. There were no statistically significant differences observed in the content of lutein and astaxanthin between the three diets.

**Figure 2:**
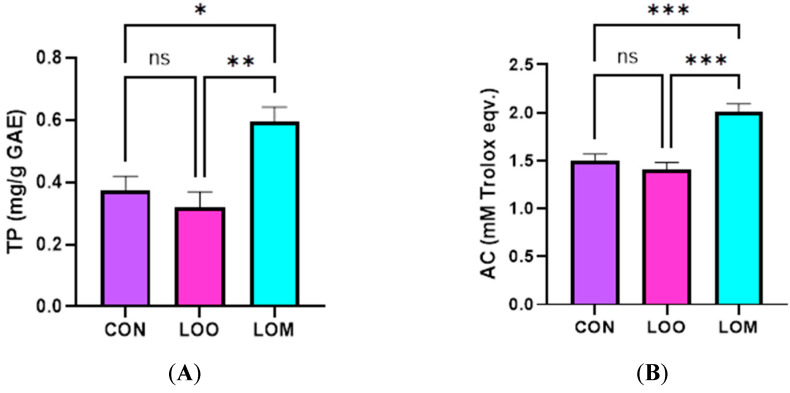
Total polyphenol content (**A**) and total antioxidant capacity (**B**) of milk. CON—control group; LOO—oxidized linseed oil group; LOM—oxidized linseed oil and milk thistle cake group; ns—not significant; * *p* < 0.05; ** *p* < 0.01; *** *p* < 0.0001. The errors bars represent the standard deviations (N = 30; n = 10).

Considering the hydro-soluble antioxidant compounds, the total polyphenol content and total antioxidant capacity were also influenced by the experimental diets (Figure 2A,B). As shown in Figure 2A, the content of total polyphenols decreased when adding the oxidized linseed oil to the goats’ diets. At the same time, the LOM group showed elevated concentrations of the total polyphenols compared with the LOO diet (*p* = 0.014). The same influence was also observed in the case of the total antioxidant capacity, which was significantly increased in the LOM group.

### 3.6. Lipid Degradation Parameters in Goat Milk

The influence of the dietary treatment on the milk’s unsaturated fatty acid concentration increased lipid oxidation susceptibility as a consequence of an imbalance between the pro-oxidative compounds and antioxidants. The primary indicators of fatty acid degradation (conjugated dienes and trienes), considering three different storage times (t0—fresh time, t1—five days of depositing, t2—ten days of depositing) at room temperature (25 °C), are reported in Table 7. Dietary treatment influenced both concentrations of CDs and CTs. In the case of CDs, it was observed that the oxidized linseed oil treatment led to a statistically significant increase in concentration compared with the CON. The same trend was also observed for the LOM diet, which expressed higher CD values than the CON group. On the other hand, the LOM group presented a significantly lower value than the LOO group, which can be attributed to the inclusion of milk thistle cake in the diet. Moreover, the content of CTs increased for the LOO diet, while the LOM did not exert a significant influence on the content, but there was a numerical decrease compared with the LOO group.

To correctly appreciate the state of milk lipid degradation, the secondary parameters of lipid oxidation were studied. The TBARS parameters and p-anisidine concentration of milk, studied at three different storage times (t0—fresh time, t1—five days of depositing, t2—ten days of depositing) at room temperature (25 °C), are reported in Table 8. The experimental diet influenced the TBARS parameters overall by increasing their levels (absorbances read at 450 nm, 495 nm, 532 nm, and MDA concentrations) for the LOO and LOM diets. However, the LOM group showed a numerically lower value for the TBARS parameters compared with the LOO group. In contrast, there was a significant increase in p-anisidine values in the group fed oxidized linseed oil, while the inclusion of milk thistle cake in the diet led to a significant decrease in its value compared to both CON and LOO groups. In the case of depositing time, the TBARS parameters and p-anisidine values showed significant increases with the extension of the storage time.

## 4. Discussion

### 4.1. Oxidation State of Oils Tested in the Experiment

Considering that this study aimed to determine the effects of natural antioxidants from milk thistle cake to counteract the effects of oxidized fats from the goats’ diet, the analysis of the oils’ oxidation state was imperative. The results showed that the oxidized linseed oils had significantly higher values than the fresh linseed oil (*p* = 0.001). The PVs of both oils were found to be within the acceptable range established by the Codex Alimentarius, FAO/WHO [37], which specifies that the maximum permissible PV for edible vegetable oils is less than 15 meq O_2_/kg. In this study, the maximum PV for the oxidized linseed oil was 12.42 meq O_2_/kg, which makes it safe for consumption.

### 4.2. Proximate, Mineral, and Lipid Composition and Bioactive Compounds of Milk Thistle Cake

Milk thistle (*Silybum marianum*) has a high protein content with a composition rich in exogenous amino acids, as confirmed in [38]. These amino acids not only support maintenance and growth, but also enhance milk protein synthesis, potentially compensating for oxidative-stress-related disruptions in metabolic pathways. The proximate chemical composition aligns with the existing literature, where the composition of total protein is reported as 23.08–21.99% DM and crude fat as 2.62–7.48% DM [27].

The mineral levels align with those reported in the literature [39]. However, the variability in the results can be explained by the differences in the varieties of plants and the soil components, which are known factors that can have an important influence on the plant’s trace mineral composition [40]. The fat from milk thistle cake has a remarkable profile of fatty acids, with oleic and linoleic acids being the main components, which is in line with previously published data [41,42,43]. In addition, the literature highlighted that milk thistle cake can be a rich source of oleic acid (60.3–49.8%) and linoleic acid (28.5–19.7%), which is in line with the data obtained in our study [27]. The variation in the composition of fatty acids can also be influenced by the species of milk thistle used and the extraction method [27]. These fatty acids are important in the synthesis of omega-6 fatty acids, being crucial in the maintenance of human health [44]. The high concentration of oleic acid in milk thistle cake can have potential benefits for ruminants, as studies have demonstrated that it can have positive effects in the reduction of methane emissions and can contribute to an increase in the CLA levels in milk [45].

The liposoluble antioxidant compounds revealed that milk thistle cake is an important source of tocopherol, with α-tocopherol being the predominant isomer. Although the literature regarding the studies on the tocopherol isomers in milk thistle cake is scarce, the data reported on milk thistle seed oil are comparable with these findings [46]. Moreover, the milk thistle cake was shown to have important concentrations of total polyphenols and total antioxidant capacity, making it a good antioxidant source to counteract lipid oxidation, aligning with data presented in another study [45]. The literature regarding the antioxidant potential of milk thistle cake is scarce. Maaloul et al. presented data from a DPPH analysis in the range of 6.595 and 2.009 mg Trolox equivalents for milk thistle seed oil, while Boško et al. presented results in the range of 13.39–34.15 mg ascorbic acid equivalents, highlighting the important antioxidant potential of milk thistle [27,45]. The individual profile of polyphenols in milk thistle cake showed a high content of flavonoids, with epicatechin being the most abundant. These findings are significant regarding antioxidant potential, as it has been shown that the antioxidant capacity is mainly correlated with flavonoid concentrations [45]. The literature regarding the individual polyphenol content of milk thistle presented contradictory results due to the high variability in plant genotypes and growth conditions [45,47]. It is also worth noting the high content of chlorogenic acid, as this has also been identified by other researchers in important amounts [47]. Although numerous studies have focused on determining the flavonolignans (silybin, isosilibinin, silidianin, and silicristin) [45], other polyphenols, such as chlorogenic acid, are very important, particularly due to their powerful antioxidant and antibacterial properties [19]. This aspect is significant in the content of potential oxidations of feeds with a high amount of fats.

### 4.3. Effect of Dietary Treatments on Proximate and Mineral Composition of Goat Milk

In goats fed oxidized linseed oil supplemented with milk thistle cake (LOO and LOM groups), the milk fat, protein, and lactose content did not differ significantly when compared with the CON milk samples (*p* > 0.05), which is consistent with existing literature data [3,48]. However, a significant reduction was observed in the casein fraction (*p* < 0.05), suggesting a selective impact on protein partitioning rather than total protein synthesis. This observation aligns with the findings of [49], who reported that dietary oxidized lipids can impair milk casein content through oxidative damage to mammary epithelial cells. In addition, the literature showed that even milk thistle demonstrated a higher crude protein degradation rate in the rumen than other feedstuffs such as soybean meal, exhibiting lower digestibility in the small intestine. This is a crucial factor, as the small intestine absorbs significant proportions of macronutrients such as proteins. Thus, the reduction in nutrient digestibility in the small intestine may limit the availability of the essential nutrients—mainly amino acids—required for milk casein synthesis [48]. However, the inclusion of milk thistle cake in the goats’ diet may have provided hepatoprotective and antioxidative effects sufficient to preserve the overall protein output, possibly via the action of silymarin compounds. These results align with the findings reported by [50], who reported antioxidant effects of silymarin that preserve lactoprotein synthesis under dietary oxidative stress.

In terms of the mineral content, the inclusion of milk thistle cake led to a significant increase in the iron content of milk. This enhancement can be attributed to the effects of milk thistle, which can help the absorption of minerals due to its fiber content. The milk thistle cake cellulose content was reported in the range of 11.92 to 26.40 g/100 g fresh sample, hemicellulose from 2.74 to 5.32 g/100 g fresh sample, and lignin from 13.27 to 18.29 g/100 g fresh sample, resulting in the total insoluble fiber ranging from 30.41 to 49.44 g/100 g fresh sample, as reported in [51]. Similarly, the authors in [52] reported that dietary fibers contain 42.9% cellulose, 21.2% hemicellulose, and a low lignin content in the flower heads of milk thistle. This highlights the fact that the content of cellulose, hemicellulose, and lignin can vary between milk thistle cake and flower heads, which can significantly affect mineral binding and bioavailability. The available studies in the literature reported that several cellulose fractions (semi-purified insoluble and soluble fractions) possess mineral-binding activity and their absorption can be enhanced at the intestinal level, which may explain the high level of iron in the milk from the LOM group [53]. Studies reported that the trace mineral content in milk can also be influenced by the minerals’ supplementation in the goats’ diet [44]. The milk’s phosphorus content was also significantly increased by the LOO and LOM diets (*p* = 0.031). There are no available data regarding the influence of oxidized linseed oil and milk thistle cake on milk’s phosphorus content. However, a study performed by Klop et al. [54] demonstrated a positive correlation between phosphorus, lactose, and casein in milk [45]. In this study, the lactose content was not statistically influenced by the experimental diets, but a numerical increase in content in the LOM diet (+3.85%) was observed, and the content of casein was decreased by the milk thistle cake. These micronutrients are essential for consumers. Phosphorus is involved in supporting bone development and energy metabolism, while iron plays a crucial role in hemoglobin synthesis and anemia prevention [55]. Further research is therefore required to clarify how milk thistle cake influences milk phosphorus content and its relationship with lactose and casein.

### 4.4. Effect of Dietary Treatments on Lipid Composition of Goat Milk

The lipid profile of goat milk was also influenced by the experimental diets. As presented in Table 5, the SFA content of the milk from the LOO samples significantly increased (*p* = 0.005), while the contents of PUFA and MUFA decreased (*p* = 0.025; *p* = 0.013). This can occur as a consequence of linseed oil oxidation. During the oxidation process, the levels of linolenic acid, which can be found in high amounts in fresh linseed oil, decreased as a consequence of this process. In the milk samples, the PUFA content presented a significant decrease, while the SFA concentration increased [56]. Our results also revealed a significant decrease in alpha-linolenic acid in both the LOO and LOM groups—results that can be attributed to the effects of including oxidized linseed in the diet. However, the inclusion of milk thistle cake along with the oxidized linseed oil resulted in a mitigation of these effects, as the concentration of SFAs, MUFAs, and PUFAs were not significantly influenced when compared with the CON samples. Similarly, Karaiskou et al. [19] reported an improvement in milk’s fatty acid quality (decreased SFAs and increased PUFAs and MUFAs) by including only milk thistle oil in the ewe diet (3% *w*/*w*). Similarly, Shedeed et al. [57] reported that silymarin-rich extract levels (10, 20, and 30 g/day) from milk thistle significantly decreased SFA content (67.3 to 68.46 vs. 69.82) but increased the MUFA (30.48 to 31.60 vs. 29.13) and PUFA (1.06 to 1.17 vs. 1.05) content in goat milk. However, the reported results for PUFA content in milk are lower when compared with our results (8.76 in the LOO group and 9.26 in the LOM group), which show that linseed oil is efficient in improving the fatty acid content than milk thistle alone. These results are significant in terms of consumers’ health. High levels of SFA can increase the risk of several health issues, mainly cardiovascular diseases, while PUFAs contribute to cardiovascular diseases, diabetes, and atherosclerosis. Moreover, the diet that included milk thistle cake led to an increase in conjugated linoleic acid (CLA) (*p* = 0.008), which is in accordance with data presented in the literature [19]. Shedeed et al. [57] reported that the highest value of CLA was observed with 20 g silymarin-rich extracts/day, as a response to the elevated levels of polyphenols from the antioxidant source used. Milk thistle cake is rich in linoleic acid (C18:2 n-6), as presented in Table 2, a PUFA that serves as a key precursor for the endogenous synthesis of CLA in ruminants. In the rumen, linoleic acid undergoes microbial biohydrogenation, during which intermediates such as cis-9 and trans-11 CLA are formed. Some of the linoleic acid escapes complete hydrogenation and is absorbed as CLA or vaccenic acid (trans-11 C18:1), which is then desaturated in the mammary gland by the action of Δ9-desaturase (stearoyl-CoA desaturase) to produce additional CLA [57]. Therefore, the increased linoleic acid content in milk thistle cake increases the availability of the substrate for both the ruminal and mammary pathways, leading to increased CLA concentrations in goat milk [58]. This is significant for human consumption, as CLA has several benefits for human health, such as the prevention of atherosclerosis, hypertension, diabetes, and several types of cancers [59].

### 4.5. Effect of Dietary Treatments on Antioxidant Potential of Goat Milk

The inclusion of milk thistle cake in the diet of goats receiving oxidized linseed oil exerted a protective effect on the antioxidant status of the resulting milk. The inclusion of milk thistle cake in the goats’ diet led to an increase in vitamin E content, especially in the case of alpha-tocopherol and gamma-tocopherol, compared with the LOO samples. Additionally, the concentration of vitamin A increased in the LOM group compared with the LOO group. Although the literature regarding the effects of milk thistle cake on the milk vitamin profile is scarce, one study reported a significant increase in retinol and tocopherol content after the inclusion of milk thistle in dairy goats’ diet [60]. The mechanism of tocopherol absorption in the small intestine can be related to the fatty acid profile, as the absorption can be increased by the higher content of PUFA and MUFA [61]. Moreover, the content of fat was positively correlated with the concentration of vitamin A in the milk [60]. Although previous studies have not directly measured the alpha-tocopherol in goat milk following milk thistle cake supplementation, the antioxidant effect of silymarin may help preserve vitamin E in milk by reducing the oxidative degradation of lipids under the effect of dietary stress [61].

In terms of hydro-soluble antioxidants, the inclusion of milk thistle in the diets led to a significant increase in the content of total polyphenols and antioxidant capacity compared with the CON and LOO diets. However, to date, there are no available studies evaluating the effects of milk thistle on the antioxidant capacity and hydro-soluble composition of milk. Nevertheless, several studies reported the benefits of by-products in milk quality. One study reported an improvement in the total polyphenol content of milk by including citrus pulp as a source of antioxidants in cows (18%) fed a diet rich in PUFAs. Besides the enrichment of milk in polyphenols, the citrus pulp also led to a decrease in SFAs as a result of its protective activity on the biohydrogenation process of highly polyunsaturated fats at the ruminal level [62]. There are relatively few studies addressing the improvement in antioxidant capacity. One study found that pregnant goats fed 10 to 30 g of milk thistle extract per head per day showed an increase in plasma antioxidant status due to the enhancement of the activity of antioxidant enzymes and improvement in plasma MDA concentrations [25]. In terms of dietary by-products, incorporating grape pomace into the diet of dairy cows improved the antioxidant capacity of the milk [63]. 

### 4.6. Effect of Dietary Treatments on Goat Milk Degradation Parameters

To the best of our knowledge, no published studies have examined the influence of milk thistle cake on the degradation parameters of milk from goats fed oxidized fats. Our results regarding the milk degradation parameters showed that the primary oxidation products (conjugated dienes and conjugated trienes) were strongly influenced by the experimental diets (Table 7). For both CDs and CTs, the levels were significantly increased by the LOO diet, while the inclusion of milk thistle cake significantly reduced the CD content of the milk. Even though there was no significant difference, a numerically decreased value was observed in the CT levels in the LOM group compared with the LOO group. In the case of the secondary oxidation products, the TBARS parameters (Table 8) were significantly increased by both the LOO and LOM diets. While not reaching statistical significance, the LOM treatment group showed a numerical reduction compared to the LOO group for the TBARS. In contrast, for the p-anisidine value, the LOM diet significantly reduced its level in milk. These results suggest that the inclusion of milk thistle cake can positively influence the milk’s primary and secondary degradation products. The increase in degradation parameters observed in the LOO group is supported by the literature, which describes an increase in the degradation parameters of milk after linseed oil post ruminal supply (75–600 mL/day) in dairy cows [64]. In terms of the secondary oxidation products, the inclusion of milk thistle extract (20 g/day and 30 g/day) in dairy cows led to a significant decrease in the content of MDA in plasma levels [25]. This contrasts with our study, but the dosage used and the specificity of the samples analyzed must be taken into consideration. However, the significant effect on milk p-anisidine can be related to the high antioxidant compounds of milk thistle, especially its remarkable content of polyphenols, which are known to be implicated in the reduction of p-anisidine levels [25]. It was also reported that polyphenols play important roles in the antioxidant potential of milk. Onjaiuea et al. described the significant impact of cyanidin-3-glucoside on milk antioxidant capacity, an anthocyanin that we also detected in an important amount for the milk thistle cake studied [65]. Studies on the inclusion of silymarin extract in ruminant diets have highlighted its powerful antioxidant capacity and protective effects against milk fat oxidation. In dairy cows, the inclusion of 7.77 g of silymarin/day/head, along with 1.27 g of lycopene, resulted in lower TBARS (thiobarbituric acid reactive substance) parameters [66]. Moreover, the inclusion of silymarin in laying hens led to improvements in the antioxidant properties of egg yolk and reduced lipid peroxidation [67].

## 5. Conclusions

The inclusion of oxidized linseed oil and milk thistle cake in dairy goats’ diet does not influence the proximate composition of the milk, with the exception of the casein level, which was decreased in the LOM group, possibly as a result of the low degradability of protein at the intestinal level. On the other hand, the iron content increased in the milk from the LOM group, which may be due to the influence of the milk thistle cake cellulose fraction that can bind iron and increase its absorption. In terms of fatty acid, the results suggested that milk thistle cake can counteract the effects of the oxidized oil by modulating the content of SFAs, MUFAs, and PUFAs, and reduce its negative effects. Additionally, the milk antioxidant potential was influenced by the experimental diets, with the LOM diet leading to an increase in the content of antioxidant compounds (vitamin E, total polyphenols, and antioxidant capacity). As a result, an improvement in the milk primary and secondary degradation parameters was observed, as the levels of conjugated dienes and p-anisidine were significantly decreased. Future studies should concentrate on optimizing the inclusion levels of milk thistle cake and ensuring feed quality to enhance these protective effects.

## Figures and Tables

**Table 1:** Ingredients and nutritional composition of experimental diets.

Ingredients in Concentrate (as Feed, %)	CON	LOO	LOM
Maize	48.4	48.4	48.4
Triticale	31.6	31.6	31.6
Sunflower meal	10.0	10.0	0.0
Linseed oil	7.0	0.0	0.0
Oxidized linseed oil	0.0	7.0	7.0
Milk thistle cake	0.0	0.0	10.0
Calcium carbonate	1.0	1.0	1.0
Sodium chloride	1.0	1.0	1.0
Mineral–vitamin supplement	1.0	1.0	1.0
Nutrients in total diet (g/day)			
DM, as fed	1926.5	1926.5	1933.1
MFU	1.6	1.6	1.7
IDPE	150.4	150.4	148.8
IDPN	159.6	159.6	151.9
Proximate composition (%)			
Dry matter	90.3	92.3	90.2
Crude protein	13.5	13.7	12.3
Crude fat	8.5	8.4	9.1
Crude fiber	5.9	6.2	6.3

CON—Control group; LOO—oxidized linseed oil group; LOM—oxidized linseed oil and milk thistle cake group; DM—dry matter; MFU—milk feed units; IDPE—intestinally digestible protein allowed by rumen-available energy; IDPN—intestinally digestible protein allowed by rumen-available nitrogen. Note: 1 kg of premix contains 0.5 g vitamin A 1000.000 IU/g, 0.3 g vitamin D3 500.000 IU/g, 4 g vitamin E 500 IU/g, 14.5 g manganese oxide 62%, 21.5 g ferrous sulfate 28%, 7.5 g zinc oxide 80%, 1.4 g sodium selenite 4.5%, and 15.3 g calcium carbonate.

**Table 2:** Proximate, mineral, and lipid composition of milk thistle cake (n = 3).

Parameter		Milk Thistle Cake
Proximate composition (%)		
Dry matter		92.48 ± 1.21
Crude protein		20.58 ± 0.95
Crude fat		8.18 ± 0.88
Crude fiber		27.84 ± 1.12
Mineral composition		
Copper (µg/g)		27.15 ± 1.37
Iron (µg/g)		129.3 ± 1.34
Manganese (µg/g)		55.15 ± 1.49
Zinc (µg/g)		90.96 ± 1.55
Calcium (%)		0.91 ± 0.098
Phosphorus (%)		0.84 ± 0.13
Lipid composition (g/100 g)		
Miristic acid	C 14:0	0.06 ± 0.001
Palmitic acid	C 16:0	9.41 ± 0.198
Palmitoleic acid	C 16:1	0.05 ± 0.002
Stearic acid	C 18:0	4.85 ± 0.163
Oleic acid	C 18:1	33.68 ± 0.148
Linoleic acid	C 18:2n6	47.77 ± 0.127
α-linolenic acid	C 18:3n3	0.23 ± 0.021
Octadecatetraenoic acid	C18:4n3	2.76 ± 0.158
Eicosadienoic acid	C 20:2n6	0.51 ± 0.021
Docosadienoic acid	C22:(2n6)	0.46 ± 0.020
Other fatty acids		0.30 ± 0.018
ΣSFA		14.31 ± 0.362
ΣMUFA		33.72 ± 0.150
ΣPUFA		51.72 ± 0.306
Σ Omega 3		2.98 ± 0.178
Σ Omega 6		48.73 ± 0.127
Omega 6/Omega 3		16.34 ± 0.936

ΣSFA—total content of saturated fatty acids; ΣMUFA—total content of monounsaturated fatty acids; ΣPUFA—total content of polyunsaturated fatty acids.

**Table 3:** The content of vitamin E isomers, lutein, total polyphenols, total antioxidant capacity, and individual polyphenols in milk thistle cake (n = 3).

Parameters	Milk Thistle Cake
Antioxidant potential	
α-tocopherol (mg/kg)	85.75 ± 1.484
γ-tocopherol (mg/kg)	6.55 ± 0.919
δ-tocopherol (mg/kg)	4.90 ± 0.565
Total vitamin E (mg/kg)	97.20 ± 1.838
Lutein (mg/kg)	2.48 ± 1.435
Total polyphenols (mg/g GAE)	4.79 ± 0.265
DPPH (mM equiv. Trolox)	2.45 ± 0.135
Individual polyphenols (mg/kg)	
*Phenolic acids*	
Gallic Acid	72.75 ± 1.343
Vanillic acid	12.75 ± 1.340
Caffeic acid	34.75 ± 1.484
Syringic acid	298.90 ± 13.717
Hydroxybenzoic acid	49.90 ± 13.838
Protocatechuic acid	280.45 ± 1.484
Chlorogenic acid	444.85 ± 14.637
Ferulic acid	232.75 ± 13.930
Methoxy cinnamic acid	13.17 ± 1.449
Trans Cinnamic acid	4.55 ± 1.485
Ellagic acid	33.65 ± 1.626
Coumaric acid	10.44 ± 1.491
*Flavonoids*	
Catechin	54.40 ± 1.697
Epigallocatechin	452.75 ± 4.454
Epicatechin	873.75 ± 14.919
Rutin	371.85 ± 13.228
Quercetin	7.25 ± 1.343
*Stilbenes*	
Resveratrol	215.40 ± 1.556
*Anthocyanin*	
Cyanidine-3-glucoside chloride	21.85 ± 1.626
Total polyphenols	3486.17 ± 88.996

GAE—gallic acid equivalents; DPPH—2,2-diphenyl-1-picrylhydrazyl-hydrate method.

**Table 4:** Proximate and mineral composition of goat milk, and milk production (N = 30; n = 10).

Parameters	CON	LOO	LOM	SEM	*p*
Milk production					
Milk (mL)	1017.85	1025.43	1015.43	85.12	0.995
Proximate composition (%)					
Fat	5.36	5.49	4.60	0.315	0.119
Protein	4.87	4.90	4.33	0.180	0.054
Casein	43.20 a	43.17 a	38.26 b	1.506	0.041
Lactose	4.94	4.93	5.12	0.095	0.273
Mineral content					
Iron (mg/kg)	13.24 b	14.06 a,b	14.93 a	0.404	0.025
Zinc (mg/kg)	42.66	41.32	43.23	2.731	0.880
Calcium (%)	1.03	1.06	1.04	0.018	0.328
Phosphorus (%)	1.062 b	1.087 a	1.086 a	0.007	0.031

CON—control group; LOO—oxidized linseed oil group; LOM—oxidized linseed oil and milk thistle cake group; SEM—standard error of the mean; a,b Means with no common letters statistically differ at *p* < 0.05, according to the ANOVA test; *p*—*p* value.

**Table 5:** Effect of dietary treatments on the lipid profile of goat milk (N = 30; n = 10).

Fatty Acids (g/100 g)		CON	LOO	LOM	SEM	*p*
Butyric acid	C 4:0	0.061	0.068	0.054	0.005	0.153
Caproic acid	C 6:0	2.750	2.728	2.755	0.113	0.647
Nonanoic acid	C 9:0	0.222	0.216	0.272	0.027	0.286
Capric acid	C 10:0	10.174	10.426	10.456	0.443	0.882
Undecanoic acid	C 11:0	0.485	0.392	0.486	0.046	0.280
Lauric acid	C 12:0	0.332	0.229	0.298	0.056	0.650
Miristic acid	C 14:0	9.729 b	10.921 a	10.785 a,b	0.331	0.031
Miristoleic acid	C 14:1	0.752	0.640	0.841	0.059	0.078
Pentadecanoic acid	C 15:0	0.234	0.242	0.258	0.013	0.400
Pentadecenoic acid	C 15:1	1.304	1.181	1.427	0.088	0.175
Palmitic acid	C 16:0	19.717 b	23.355 a	21.314 b	0.506	<0.0001
Palmitoleic acid	C 16:1	2.120 a	1.351 b	2.120 a	0.103	<0.0001
Heptadecanoic acid	C 17:0	0.661	0.640	0.703	0.039	0.534
Heptadecenoic acid	C 17:1	0.356	0.300	0.331	0.020	0.159
Stearic acid	C 18:0	6.111	6.689	5.684	0.459	0.328
Cis-oleic acid	C18:1n9c	25.200	22.784	23.743	0.784	0.105
Trans-linoleic acid	C18:2n6t	3.935	3.359	3.504	0.251	0.251
Cis-linoleic acid	C18:2n6c	4.243	3.431	3.783	0.306	0.185
Arachidic acid	C20:0	0.269	0.162	0.231	0.023	0.102
Gamma-linoleic acid	C18:3n6	0.248	0.117	0.150	0.041	0.080
Alpha-linolenic acid	C18:3n3	1.700 a	1.340 b	1.258 b	0.061	<0.0001
Conjugated linoleic acid	CLA (c9, t11)	0.546 a,b	0.417 b	0.746 a	0.067	0.008
Octadecatetraenoic acid	C18:4n-3	0.195	0.140	0.142	0.017	0.050
Eicosadienoic acid	C20:2n6	0.119	0.074	0.154	0.024	0.086
Eicosatrienoic acid	C20:3n6	0.205	0.156	0.192	0.048	0.764
Arachidonic acid	C20:4n6	0.158	0.142	0.083	0.031	0.233
Other fatty acids		0.736	0.656	0.669	0.050	0.473
ΣSFA		58.11 b	63.88 a	61.06 a,b	1.123	0.005
ΣMUFA		29.80 a	26.28 b	28.26 a,b	0.783	0.013
ΣPUFA		10.80 a	8.76 b	9.26 a,b	0.521	0.025
Omega 3		2.02 a	1.40 b	1.40 b	0.062	<0.0001
Omega 6		9.45	7.69	8.61	0.543	0.090
Omega 6/Omega 3		5.03	5.28	6.22	0.383	0.089
Cholesterol		0.018	0.018	0.016	0.001	0.117

CON—control group; LOO—oxidized linseed oil group; LOM—oxidized linseed oil and milk thistle cake group; SEM—standard error of the mean; ΣSFA—total content of saturated fatty acids; ΣMUFA—total content of monounsaturated fatty acids; ΣPUFA—total content of polyunsaturated fatty acids; a,b Means with no common letters statistically differ at *p* < 0.05, according to the ANOVA test, *p*—*p* value.

**Table 6:** Effect of dietary treatments on the lipo-soluble antioxidant compounds in goat milk (N = 30; n = 10).

Parameters (mg/kg)	CON	LOO	LOM	SEM	*p*
α-tocopherol	28.18 a	13.35 b	27.81 a	0.220	<0.0001
γ-tocopherol	2.08 b	2.04 b	2.75 a	0.108	<0.0001
δ-tocopherol	1.75	1.70	1.72	0.020	0.241
Total vitamin E	31.93 a	17.17 b	32.28 a	0.334	<0.0001
Vitamin A	4.66 a	2.02 c	3.47 b	0.290	<0.0001
Lutein	0.340	0.360	0.341	0.023	0.783
Astaxanthin	1.47	1.49	1.59	0.114	0.742

CON—control group; LOO—oxidized linseed oil group; LOM—oxidized linseed oil and milk thistle cake group; SEM—standard error of the mean; a,b,c Means with no common letters statistically differ at *p* < 0.05, according to the ANOVA test; *p*—*p* value.

**Table 7:** Primary degradation parameters of milk at different storage times (N = 30; n = 10).

Storage Time	Dietary Treatment	CD (mL/g)	CT (mL/g)
t0	CON	30.38	1.78
	LOO	45.11	2.37
	LOM	36.22	1.78
t1	CON	29.52	2.06
	LOO	48.17	2.74
	LOM	45.68	2.47
t2	CON	26.53	2.31
	LOO	46.86	3.49
	LOM	38.91	2.54
Statistical analysis			
*Dietary treatment*			
CON		28.81 c	2.05 b
LOO		46.72 a	2.87 a
LOM		40.27 b	2.26 a,b
SEM		1.77	0.207
*Storage time*			
t0		37.24	1.98 b
t1		41.13	2.42 a,b
t2		37.44	2.78 a
SEM		1.78	0.207
*p* values			
Diet		0.004	0.023
Storage time		0.264	0.035
Diet x Storage time		0.521	0.848

CD—milk conjugated diene concentration; CT—milk conjugated triene concentration; t0—fresh milk samples; t1—five-day milk storage time; t2—ten-day milk storage time; CON—control group; LOO—oxidized linseed oil group; LOM—oxidized linseed oil and milk thistle cake group; SEM—standard error of the mean. a,b,c Means with no common letters statistically differ (*p* < 0.05); *p*—*p* value.

**Table 8:** TBARS parameters and concentration of p-anisidine in milk at different storage times (N = 30; n = 10).

Storage Time	Dietary Treatment	450 nm	495 nm	532 nm	MDA, µG/L	p-Anisidine (A.U.)
t0	CON	0.301	0.145	0.116	92.36	25.01
	LOO	0.343	0.179	0.148	134.97	27.14
	LOM	0.323	0.171	0.139	127.31	23.29
t1	CON	0.372	0.188	0.157	148.07	76.67
	LOO	0.391	0.214	0.179	176.99	94.60
	LOM	0.397	0.209	0.180	177.72	68.01
t2	CON	0.404	0.196	0.161	145.29	94.03
	LOO	0.445	0.240	0.197	186.32	104.113
	LOM	0.441	0.230	0.192	182.09	73.29
Statistical analysis						
*Dietary treatment*						
CON		0.359 b	0.176 b	0.145 b	128.57 b	65.24 a
LOO		0.393 a	0.211 a	0.174 a	166.09 a	75.28 a
LOM		0.387 a	0.203 a	0.170 a	162.37 a	54.86 b
SEM		0.006	0.004	0.003	4.514	2.873
*Storage time*						
t0		0.322 c	0.165 c	0.134 b	118.21 b	25.14 c
t1		0.387 b	0.204 b	0.172 a	167.59 a	79.76 b
t2		0.430 a	0.222 c	0.183 a	171.24 a	90.48 a
SEM		0.004	0.004	0.003	4.521	2.875
*p* values						
Diet		0.002	0.002	<0.0001	<0.0001	<0.0001
Storage time		<0.0001	<0.0001	<0.0001	<0.0001	<0.0001
Diet × Storage time		0.705	0.827	0.781	0.918	0.056

t0—fresh milk samples; t1—five-day milk storage time; t2—ten-day milk storage time; CON—control group; LOO—oxidized linseed oil group; LOM—oxidized linseed oil and milk thistle cake group; SEM—standard error of the mean; MDA—malondialdehyde; A.U.—arbitrary units. a,b,c Means with no common letters statistically differ (*p* < 0.05); *p*—*p* value.

## Data Availability

The original contributions presented in the study are included in the article, further inquiries can be directed to the corresponding author.
